# Searching Hit Potential Antimicrobials in Natural Compounds Space against Biofilm Formation

**DOI:** 10.3390/molecules25225334

**Published:** 2020-11-16

**Authors:** Roberto Pestana-Nobles, Jorge A. Leyva-Rojas, Juvenal Yosa

**Affiliations:** Facultad de Ciencias Básicas y Biomédicas, Laboratorio de Simulación Molecular y Bioinformática, Universidad Simón Bolivar, Barranquilla 080002, Colombia; roberto.pestana@unisimon.edu.co (R.P.-N.); jorge.leyva@unisimonbolivar.edu.co (J.A.L.-R.)

**Keywords:** biofilms, virtual screening, molecular dynamics, natural products, binding energy, *trans*-aconitic acid, hit-to-lead

## Abstract

Biofilms are communities of microorganisms that can colonize biotic and abiotic surfaces and thus play a significant role in the persistence of bacterial infection and resistance to antimicrobial. About 65% and 80% of microbial and chronic infections are associated with biofilm formation, respectively. The increase in infections by multi-resistant bacteria instigates the need for the discovery of novel natural-based drugs that act as inhibitory molecules. The inhibition of diguanylate cyclases (DGCs), the enzyme implicated in the synthesis of the second messenger, cyclic diguanylate (c-di-GMP), involved in the biofilm formation, represents a potential approach for preventing the biofilm development. It has been extensively studied using PleD protein as a model of DGC for in silico studies as virtual screening and as a model for in vitro studies in biofilms formation. This study aimed to search for natural products capable of inhibiting the *Caulobacter crescentus* enzyme PleD. For this purpose, 224,205 molecules from the natural products ZINC15 database, have been evaluated through molecular docking and molecular dynamic simulation. Our results suggest *trans*-Aconitic acid (TAA) as a possible starting point for hit-to-lead methodologies to obtain new inhibitors of the PleD protein and hence blocking the biofilm formation.

## 1. Introduction

Biofilms are communities of microorganisms that can colonize both biotic and abiotic surfaces, thus playing a significant role in the persistence of bacterial infection. Microorganisms living in biofilms are embedded within a self-produced matrix of extracellular polymeric substances (EPS) [1,2]. The extracellular matrix supports the cell-to-cell interaction in biofilms and plays an important function in several processes including cell attachment, cell-to-cell connection, structural function, and antimicrobial tolerance. This matrix produced by bacteria is mainly composed of proteins, enzymes, polysaccharides, signaling molecules, and extracellular DNA [3].

The change from free-living bacteria to biofilm involves the production of adhesins and extracellular matrix compounds that interconnect cells in biofilms [4].

This microorganism assemblage provides a protected mode of growth, with resistance to antimicrobials and killing by host immune system. The biofilm development makes the survival of pathogen microorganisms in hostile environments, as well as the dispersal and colonization of other niches possible [5].

In the medical field, biofilm phenotype has been recognized as an active agent in development of many infections, with the most common, being related to the use of medical devices [6]. The National Institutes of Health have reported that, 65% and 80% of microbial and chronic infections, are associated with biofilm formation, respectively [7]. A better understanding of the molecular and physiological mechanisms of biofilm formation will allow a tool for its inhibition.

Cyclic diguanylate (c-di-GMP) is an important second messenger involved in the biofilm organization [8]. This signaling molecule modulates bacterial growth phenotypes including, but not only limited to, biofilm formation, virulence factor production, and motility [8,9].

In the bacterial kingdom, the enzymatic GG[D/E]EF and EAL domains of diguanylate cyclase, PleD protein are highly conserved and several copies of diguanylate cyclase (DGC) proteins containing these domains are found in many bacterial genomes [10]. A consensus of all the GG[D/E]EF, EAL, and HD-GYP domains in bacterial genomes is available at http://www.ncbi.nlm.nih.gov/Complete_Genomes/c-di-GMP.html [11].

The synthesis of c-di-GMP depends on diguanylate cyclases (DGCs) which use two molecules of guanosine-5${}^{\prime }$-triphosphate (GTP) to obtain c-di-GMP in a two-step reaction; whereas phosphodiesterases (PDEs) hydrolyze c-di-GMP to linear di-GMP [9,12].

Inhibition of DGCs as a strategy for preventing biofilm formation represents a potential method and has been extensively studied using PleD protein (EC: 2.7.7.65) as a model of DGC for in silico studies (using virtual screening) and in vitro studies in biofilms formation [8,9,12,13,14].

In this work, we used as a model the DGC (PleD) from *Caulobacter crescentus*, taking into account that catalytic site of DGCs are structurally conserved (Figure S1 in in Supplementary Materialss). *Caulobacter crescentus* is a Gram-negative bacterium. It has been an important model organism, not only for investigate the regulation of the cell cycle, asymmetric cell division, and cellular differentiation but also to study the activation and synthesis of the second messenger involved in the response of the environmental adaptation [15]. The first diguanylate cyclase activity related to the synthesis of the second messenger, c-di-GMP, as a response regulator of biofilm formation, was experimentally demonstrated with *Caulobacter crescentus* [16]. During the life cycle, the bacterium produces a sessile, surface-adherent stalked cell, and a motile swarmer cell [17]. The stalked cell stage offers a fitness advantage by anchoring the cell to surfaces to form biofilms and or to exploit nutrient sources [18]. In the biofilm formation, high cellular levels of c-di-GMP promote exopolysaccharide production and surface adhesion, in contrast, in motile swarmer stages low c-di-GMP concentration result in flagellar gene expression increasing cellular motility [10]. A similar response has been observed in the human opportunistic pathogen *Pseudomonas aeruginosa*, changes in the intracellular concentration of c-di-GMP determine its physiology and pathogenesis. Thus, during the process of biofilm formation that initiates with attachment to the surface of planktonic bacteria, an increase of intracellular c-di-GMP level was observed [19].

The increase in infections due to existence of multi-resistant bacteria instigates the need for the discovery of new drugs. Bioactive molecules synthesized by microorganisms, plants, and animals, are efficient natural compounds against microbes. These substances act as chemical defense in various competitive environments [20]. An alternative for selecting DGC natural inhibitory molecules involves the search of natural products in the database, which could supply substances (especially small compounds) with good binding affinity for the target enzyme.

Since the discovery of penicillin, natural products play an important role in the discovery and development of new drugs [21,22]. Between 1981 and 2014, 43.6% of anti-infective drugs were approved and 40.7% of anticancer agents were based on natural products or derivatives thereof [22], and many natural products have become a template for drug design because they often present a ligand–protein binding motif [23]. Some natural products have shown antibacterial activity like as described by Nofiani et al. [24] and Emiru et al. [25].

**Figure 1:**
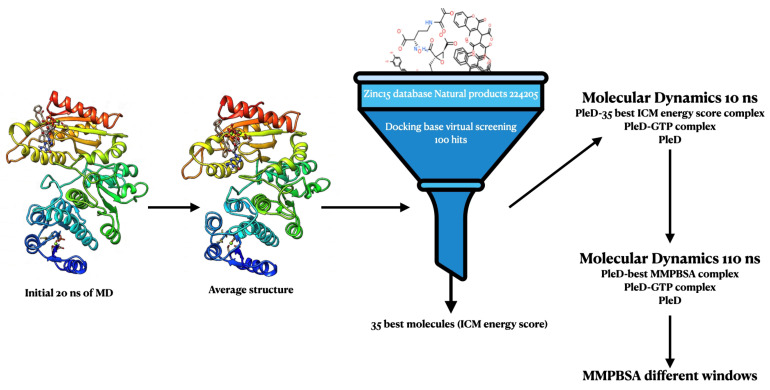
Summary of procedures used for the hit identification. An initial 20 ns of molecular dynamics (MD) was performed with the PleD protein (inactive form) in complex with guanosine-5${}^{\prime }$-triphosphate (GTP) molecule and Mg${}^{2+}$ ions, by following the crystal structure of the active form 2V0N to coupling the GTP at the catalytic site. From an equilibrated trajectory starting at 1.2 ns, an average structure was produced using chimera software [26]. A library of about 224,205 natural products compounds was obtained from ZINC15 database. Bond orders, tautomeric forms, stereochemistry, hydrogen atoms, and protonation states were assigned automatically by the ICM package, with default parameters for each compound to prepare the ligands for docking. Ligands were docked using virtual screening scheme from ICM using the PleD protein average structure obtained by the above procedure. The GTP geometric space in the protein was used as a reference for virtual screening (VS) procedure and ICM energy scoring function was used for ranking it. From the 100 hits, 35 with the best ICM energy score were selected for 10 ns of MD simulations. For each molecule, MM/PBSA was computed using 200 frames from the last 2 ns of each MD. Finally, with the best complex (Ligand4–PleD), ranked from MM/PBSA binding free energy, a 110 ns of MD was done. This was also done for the GTP–PleD protein complex and PleD protein. From the above three trajectories, MM/PBSA was computed again at different windows starting from 20 ns until 60 ns using 200 frames for each window.

In the present study, an evaluation of diguanylate cyclase, using as a model, the PleD protein from *Caulobacter crescentus* against 224,205 molecules from natural resources in ZINC15 database was carried out. Here, virtual screening (VS), molecular dynamics (MD) simulations, and binding free energy calculation adopting the Molecular Mechanics Poisson–Boltzmann Surface Area method (MM/PBSA) were used for the hit searching. A summary of the complete procedure is shown in Figure 1. This work aimed to propose potential hit substances from natural compounds database that can be optimized to generate promising lead candidate compounds as a competitive ligand for the diguanylate cyclase proteins.

## 2. Results and Discussion

Diguanylate cyclase PleD protein of *Caulobacter crescentus* belongs to the response regulator family and its activity is controlled by phosphorylation, where two cognate kinases are involved: DivJ and PleC. PleD is required for polar differentiation in the bacterial cell cycle. Bacterial cells without functional PleD are hypermobile and fail to accomplish swarmer-to-stalked cell transition [16,27,28,29]. PleD protein contains an intrinsic nucleotide cyclase activity which converts two molecules of GTP into 3${}^{\prime }$,5${}^{\prime }$-cyclic diguanylic acid (c-di-GMP) [16], a molecule of great interest which regulates surface-adhesion properties and motility in bacteria [15,30]. In addition, c-di-GMP is involved in formation and persistence of bacterial biofilm [31]. Given that c-di-GMP is exclusively found in bacteria, c-di-GMP can be a potential target for medicinal applications.

From the pseudo-active structure (PDB-ID: 2V0N), the mode of substrate binding is as far as the position of the terminal phosphates close to the P-loop, together with two Mg${}^{2+}$ ions, which are coordinated by the phosphates and two invariant carboxylates (ASP327 and GLU370). Chain A from an inactive structure (PDB-ID: 1W25) was chosen for MD and VS, and a GTP molecule and two Mg${}^{2+}$ ions were added to the catalytic site of the protein, keeping the above mentioned position of the substrate (GTP) from the pseudo-active structure. In addition, ASP52 was phosphorylated and parameters for the phosphate group were obtained following the procedure mentioned elsewhere [32]. The objective of this manuscript is to find compounds in a natural products library which compete for the active site, in such case avoiding the formation of cyclic di-GMP (c-di-GMP). The c-di-GMP second messenger represents a signaling system that regulates many bacterial behaviors and is of key importance for driving the lifestyle switch between motile loner cells and biofilm formers [19].

Activation of PleD proceeds via phosphorylation-induced dimerization. Upon modification of Asp53 of the Rec domain, the intramolecular packing of the Rec and (Rec-Rec’)2 “stem” is improved [33]. In such a way, the system was prepared to simulate a possible activation from inactive to active once molecular dynamics was run, simulating a real scenario for protein dynamics.

Molecular dynamics was done for the complex PleD–GTP for 20 ns. The monomer of PleD (chain A) was chosen and GTP molecule was added to the structure in the catalytic site, together with Mg${}^{2+}$ ions and phosphorylation at ASP52. Charges and atom types for each ligand were assigned from the gaff force field.

**Figure 2:**
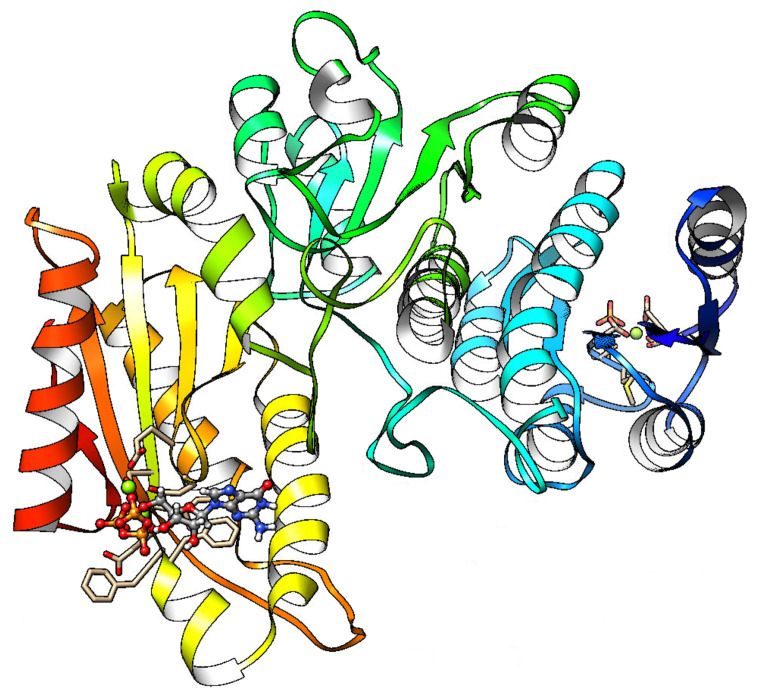
Average structure of PleD protein obtained from 20 ns of MD simulation. GTP, ASP52-phosphate moiety, and Mg${}^{2+}$ ions are presented in ball and stick representation.

The system converges at 1.2 ns of simulation (Figure S2 in Supplementary Materials). From 1.2 ns, an average structure for the rest of the trajectory was computed for VS simulation using Chimera package [26] (Figure 2). First, molecular docking was done with the GTP molecule, obtaining an ICM energy score of about −27.64 kcal/mol (Table S1 in Supplementary Materials). VS was done with natural compounds library from ZINC15, using GTP molecule from the average structure as reference. The coordinates of the ligands were extracted from the sdf file in ZINC15 natural compounds library. Bond orders, tautomeric forms, stereochemistry, hydrogen atoms, and protonation states were assigned automatically by ICM package with default parameters [34]. Atom types, charges, and parameters were obtained from Merck Molecular Force Field (MMFF) for each ligand in the VS procedure. Geometry optimization was carried out using the automatic converting procedure in ICM [34].

**Figure 3:**
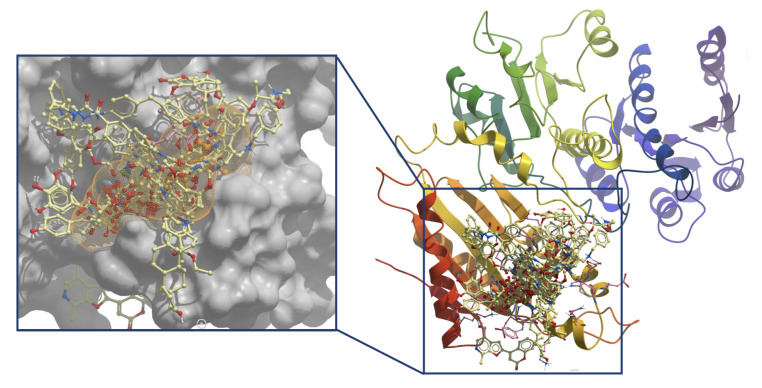
Best poses for the first 100 hits ranked according to ICM energy score. Orange dot envelope represents the space found in the average structure of GTP.

ICM scores for the best 100 hits molecules are summarized in Table S1 in Supplementary Materials. They have ICM score energies ranging from −47.13 to −31.62 kcal/mol. Based on results from the VS procedure, the first 100 natural compounds had better ICM score energy than the GTP (Figure 3). The main idea of this work is to find a compound from natural product source which can block the active site of the PleD protein, in that way, blocking the formation of c-di-GMP. To do this, a more accurate calculation of the binding free energy was done. MM/PBSA was used to compute the binding free energies for the best 35 ICM energy score molecules from VS.

### 2.1. Molecular Mechanics Poisson–Boltzmann Surface Area (MM/PBSA)

**Figure 4:**
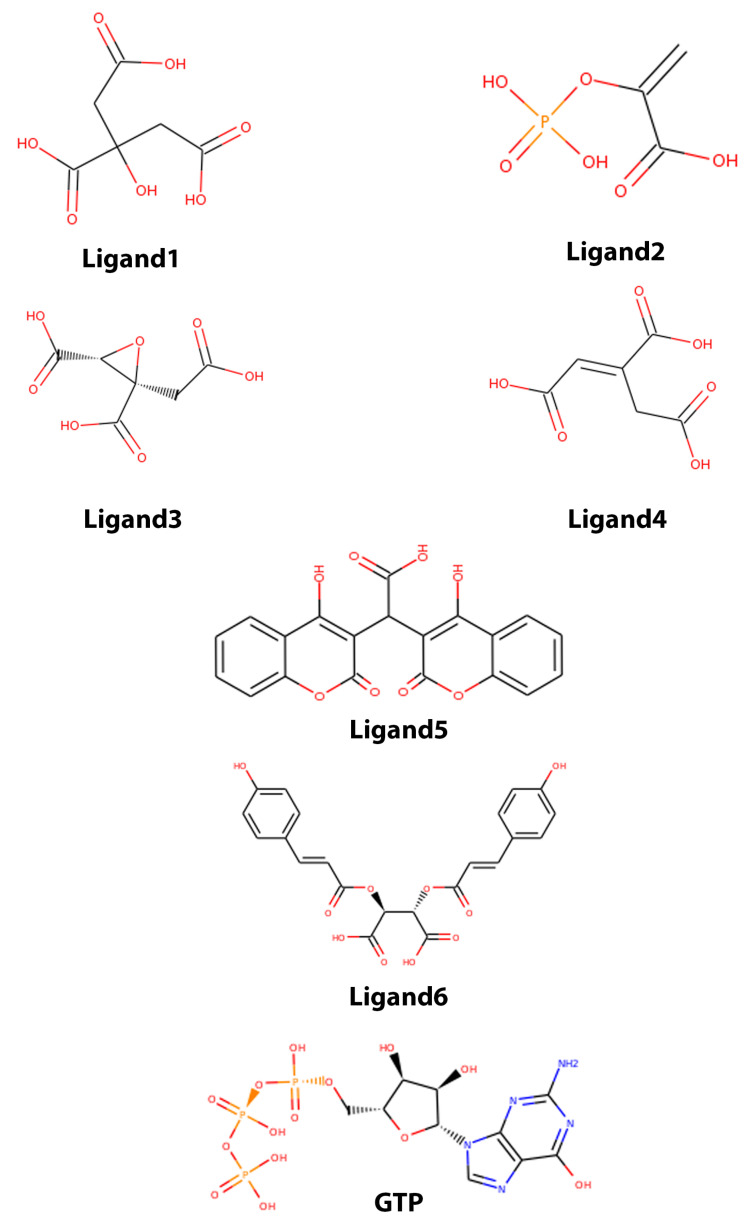
2D structure of the six ligands with best MM/PBSA performance.

The best 35 ligands from VS simulations were selected to perform 10 ns of MD simulation. From this results, the best 6 molecules with the best binding free energy MM/PBSA were studied. For the 35 ligands, the energy values were ranked between −121.39 (ZINC04501392) and −12.84 kcal/mol (ZINC15956889). The complete list is shown in Table S2 in Supplementary Materialss. On the other hand, the GTP molecule has a binding free energy of about −178.09 kcal/mol. The difference in energy from VS and MM/PBSA calculation lies, not only in the method, but the parameter and charges used for this type of calculation (see methods). The six ligands with the best binding free energy according to MM/PBSA calculations are listed in Table 1 with the ZINC15 ID. In Figure 4, a 2D structure representation of the GTP and molecules are shown. Previous studies have reported antibacterial activity using one of these ligands or derivatives of them, for instance, citrate can be used to functionalize and synthesize nanoparticles with antibacterial activity [35,36]. In searching for new nematicidal factors from *Bacillus thuringiensis* against *Meloidogyne incognita* (a plant-parasitic nematode), a component identified as trans-aconitic acid (TAA) was found. The acid showed high nematicidal activity, suggesting that TAA is specifically synthesized by the bacillus, as a virulence factor. Moreover, TAA acid has shown nematicidal activity against *Bacillus thuringiensis* bacterium [37].

The MM/PBSA calculation provides information on strength of the binding between protein and ligand. For this, the algorithm considers the binding free energy equal to the free energy of the complex minus the sum of the free energy of the protein plus the free energy of the ligand, see Equation (1).

(1) $$\Delta G=G_{complex}-\left(G_{protein}+G_{ligand}\right)$$

Each *G* component from Equation (1) can be calculated as:

(2) $$\Delta G=\langle E_{MM}\rangle -TS+\langle G_{solvation}\rangle$$

where $\langle E_{MM}\rangle$ is average molecular mechanics potential energy in vacuum; T is temperature; S is entropy; and $\langle G_{solvation}\rangle$ is the free energy of solvation. The term $\langle E_{MM}\rangle$ includes energy of both bonded and non-bonded interactions ($E_{bonded}$ and $E_{non-bonded}$). $E_{bonded}$ considers bond, angle, dihedral, and improper interactions. $E_{non-bonded}$ considers electrostatic ($E_{elec}$) and van der Waals ($E_{vdW}$) interactions, see Equation (3).

$\langle G_{solvation}\rangle$ are included polar and non-polar free energies; and polar contribution is calculated using the Poisson–Boltzmann equation while non-polar contribution includes repulsive and attractive forces between solute and solvent generated by cavity, $E_{cavity}$ formation, and van der Waals interactions $E{}_{v}dW$ see Equation (4). For more information about how the equation works and how it is calculated, consult the work of Kumari et al. [38], Baker et al. [39], Wisser et al. [40], and Konecny et al. [41].

(3) $$\langle E_{MM}\rangle =E_{bonded}+E_{non-bonded}=E_{bonded}+E_{vdW}+E_{elec}$$

(4) $$\langle G_{solvation}\rangle =G_{polar}+G_{nonpolar}=G_{polar}+E_{cavity}+E_{vdW}$$

A lower value of Δ*G* implies a better coupling between protein and ligand. Table 2 shows the values obtained for 6 ligands with the best binding free energy. Results show that none of the molecules tested had the same or close binding free energy as GTP, such that the difference between GTP and ligands energies were: Ligand1 = 78 kcal/mol; Ligand2 = 74.77 kcal/mol; Ligand3 = 58.51 kcal/mol; Ligand4 = 56.76 kcal/mol; Ligand5 = 70.16 kcal/mol; and Ligand6 = 77.11 kcal/mol. The largest difference is exhibited by Ligand1, while the lowest difference is exhibited by Ligand4. In order to have a better insight on residues that contributed more to ΔG binding free energy, energy decomposition was performed (see methodology section for details); with this, total ΔG energy is presented as a contribution per residue. Armed with this information, we can classify residues into favorable and non-favorable interactions.

**Figure 5:**
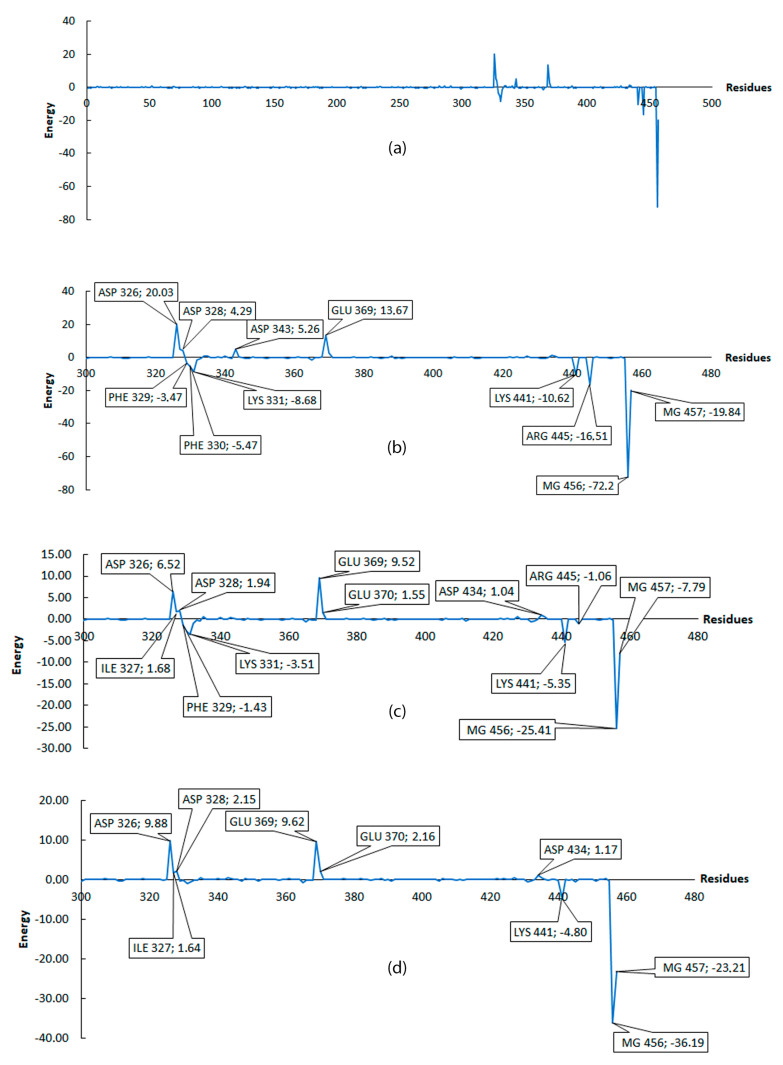
Interaction per residue, from residue 300 to 457. All energies were calculated in kcal/mol. (**a**) Contribution of all residues on MM/PBSA energy for GTP. (**b**) Zoom from residue 300 to 457 for GTP, (**c**) Ligand1, (**d**) Ligand2. Residues with contribution higher than 1 or lower than −1 are marked.

**Figure 6:**
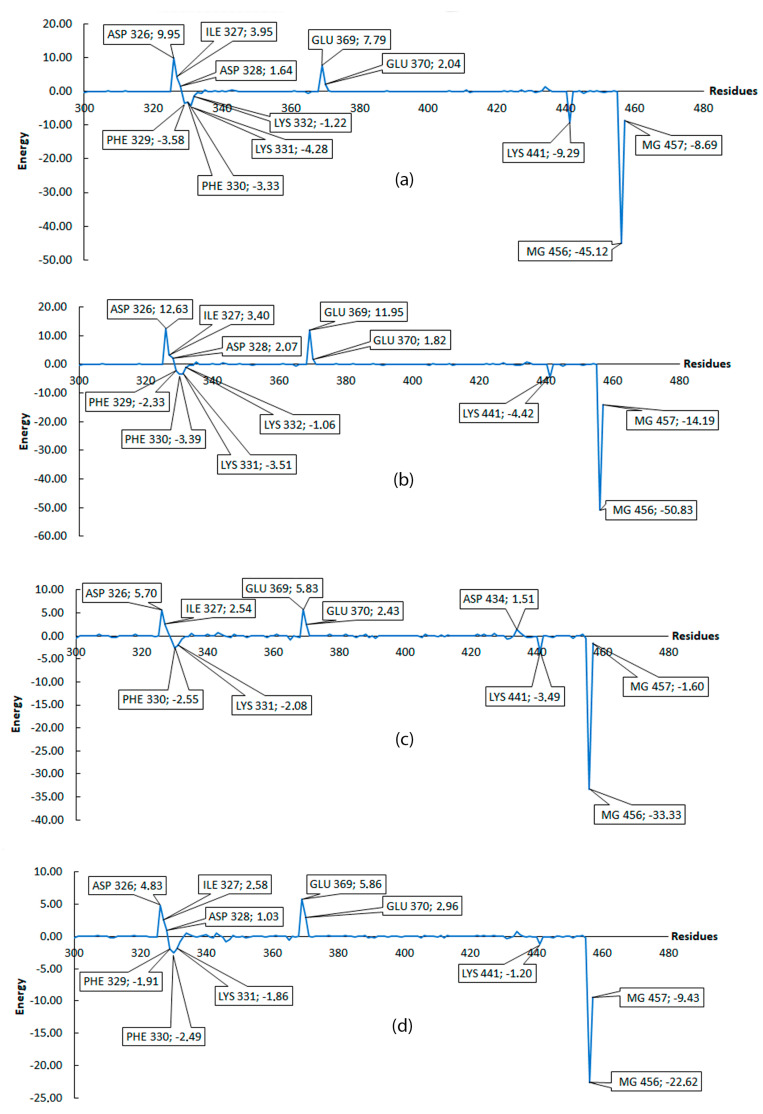
Interaction per residue from residue 300 to 457. All energies were calculated in kcal/mol. (**a**) Ligand3, (**b**) Ligand4, (**c**) Ligand5, and (**d**) Ligand6. Residues with contribution higher than 1 or lower than −1 are marked.

**Figure 7:**
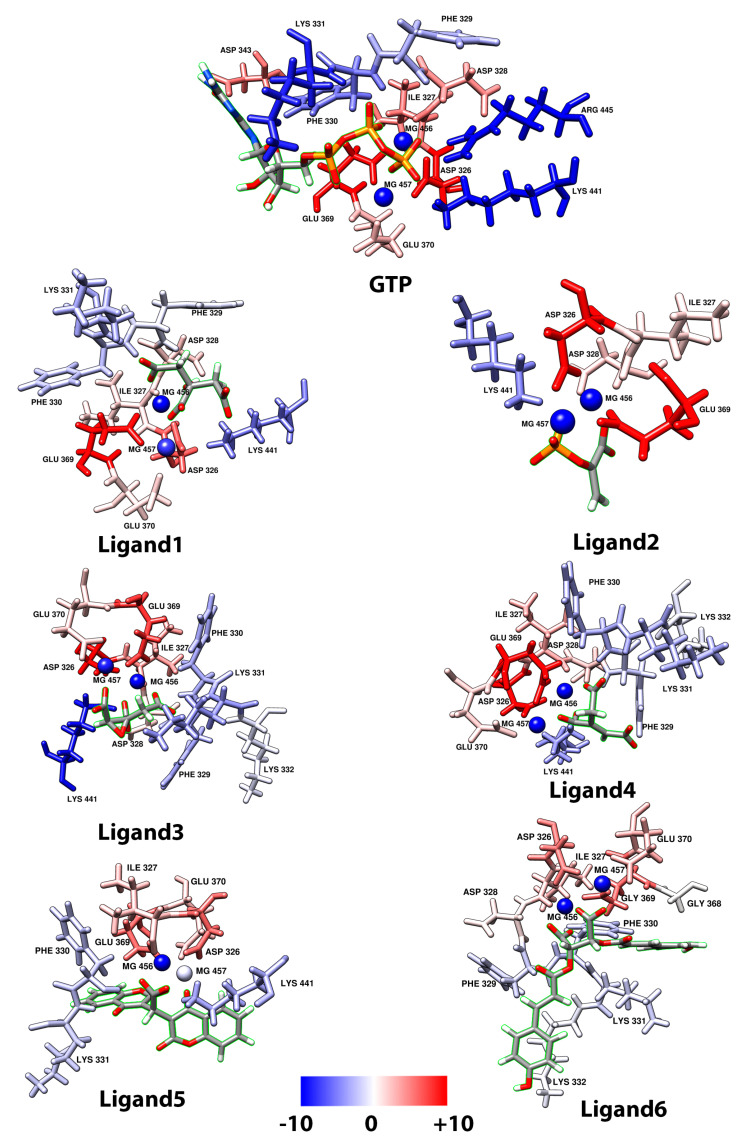
Contribution per residues around 3 Å from ligand in MM/PBSA calculation for each molecule and GTP, blue color indicates favorable interaction, meanwhile red color indicates non-favorable interaction. Ligand is highlighted in green.

Figure 5 and Figure 6 only considered residues between 300 and 457, because, as shown in Figure 5a, the first 300 residues do not contribute to the binding free energy. For Figure 5 and Figure 6, negative values are considered as favorable interactions. The interactions between two of the three magnesium present in the protein play a role in coordinating the carboxyl groups in all 6 ligands including GTP which help to gain more stability. Taking the GTP as reference, an interaction with ARG445 is observed and this is a highly favorable interaction which is not present in any of the 6 ligands evaluated, suggesting this residue as an important residue in the binding pocket which help to reach a more stable conformational configuration. The ligands show common favorable interactions with LYS331, LYS441, PHE329, and PHE330 except for Ligand2, presumably because this ligand is the shortest one, thus limiting its interaction with the protein. The result of positive energy suggest a non-favorable interaction with the protein residues, such that all ligands show non-favorable interaction with ASP326, ASP328, GLU369, ILE327, and GLU370. These five residues are common and have the higher positive values among the ligands. All ligands (except for Ligand4 and Ligand6) show non-favorable interaction with ASP434. The non-favorable interaction for GTP is mainly with ASP326 and GLU369, which is shown graphically in Figure 7.

#### 2.1.1. Root Mean Square Deviation (RMSD)

From the 10 ns molecular dynamic simulation used for the calculation of MM/PBSA, a Root Mean Square Deviation (RMSD) analysis was done. The RMSD was used to verify the protein stability during the entire simulation [42]. Less deviation in these values indicates a protein stability. For these, Table 3 shows the average RMSD for the 6 ligands and GTP.

**Figure 8:**
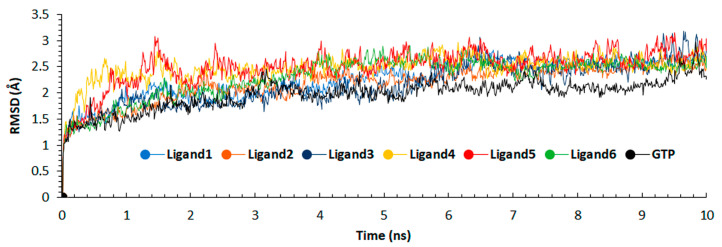
RMSD calculation for 10 ns of production for each ligand and GTP.

Figure 8 shows that the 6 ligands and GTP are stabilized around 7 ns and 4 ns, respectively. These values were obtained using cpptraj tool [43]. Aligning to the first frame of production part of the simulation, the result suggests a stable configuration of the protein with the ligands, as shown in Figure 8, where GTP shows less variation compared to the other ligands.

#### 2.1.2. Hydrogen Bonds (H-Bonds)

Hydrogen bonds represent an important interaction between protein–ligand complexes and is regarded as the main reason for protein–ligand selectivity. This is why hydrogen bond analysis is often used to describe affinity between protein and ligand [44].

With cpptraj tool the average amount of time at which a hydrogen bond was present during the simulation were analyzed. From this information, the protein residues with H-bond formation respect to the ligands are computed. For these, the H-bond analysis for each ligand (including GTP) was done using a threshold of 3 Å around the ligands during the production, and for each frame.

Among all ligands evaluated, LYS441 was the most important residue in the H-bond formation. In some cases, it is present in more than the 80% of the production (Ligand2). Residues PHE331, PHE330, and LYS331 were also present as relevant. For GTP, the residue with more interactions was ARG445, and this was also seen in MM/PBSA decomposition results.

A second analysis was performed to observe the behavior of the best ligand obtained in MM/PBSA calculation, which is Ligand4. For this, 100 ns of MD with the same condition of the previous 10 ns were done. The starting point for this new simulation was the ending of the previous one, giving a total of 110 ns of simulation. In this second analysis, the PleD–protein without any ligand (to serve as a reference), complex PleD–GTP, and complex PleD–Ligand4 were taken for simulations. MM/PBSA calculations done using different time windows 20, 30, 40, 50, and 60 ns of the molecular simulation were consider. In each window, the same amount of snapshots (200) were taken.

### 2.2. Trans-Aconitic Acid (Ligand4)

#### 2.2.1. Root Mean Square Deviation

**Figure 9:**
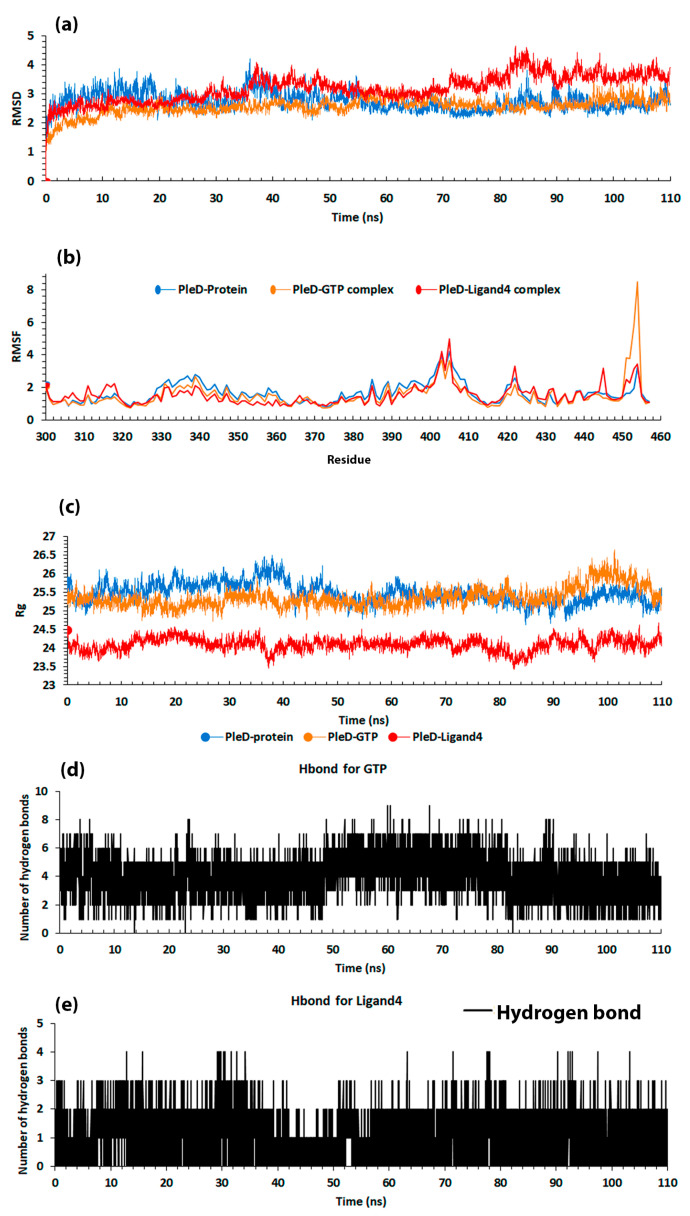
Analysis results from the second production of 110 ns, (**a**) RMSD in angstrom, (**b**) RMSF in angstrom, (**c**) Radius of gyration (Rg) in angstrom, (**d**) Hydrogen bonds for GTP, and (**e**) Hydrogen bonds for Ligand4.

RMSD showed high stability in the three cases (PleD, GTP, and Ligand4). In Figure 9a, the average value with its standard deviation were as follows: PleD–protein = 2.75 ± 0.31; PleD–GTP complex = 2.57 ± 0.28; and PleD–Ligand4 complex = 3.20 ± 0.45. The PleD–protein had a higher value than PleD–GTP, thus indicating that GTP helped the protein itself to reach a more stable configuration. The PleD–Ligand4 shows more variation, although it has high stability during the dynamic as seen in the low standard deviation.

#### 2.2.2. Root Mean Square Fluctuation

For RMSF, the same approach as MM/PBSA was made. The graph considered residues 300–457, in which the interactions where establish (Figure 9b). The same fluctuation was observed in the protein structure for the three cases, specifically in PleD–GTP. A major variation is present in residues 450–455 and the average variation and its standard deviation were: 1.65 ± 0.63 for PleD–protein; 1.53 ± 0.87 for PleD–GTP complex; and 1.53 ± 0.62 for PleD–Ligand4 complex. The RMSF analysis agrees with RMSD, in which PleD alone presents lower stability compared to its binding PleD with GTP.

#### 2.2.3. Radius of Gyration

The radius of gyration (Rg) can give an insight about the compactness of a protein structure [45]. Figure 9c suggests a structural stability in the 3 systems considered; however, the complex PleD–Ligand4 had less Rg compared to the other two structures. The average value and its standard deviation for the three systems were: 25.48 ± 0.27 for PleD–protein; 25.36 ± 0.26 for PleD–GTP; and 24.1 ± 0.17 for PleD–Ligand4.

#### 2.2.4. Hydrogen Bond (H-Bond)

The H-bond analysis results for 110 ns production show some differences compared to the results for 10 ns (Table 4 and Table 5), in which GTP was observed to have the same tendency as the previous result, where residue ARG445 is much more present during the simulation. In this case, the order is maintained, such that LYS331, LYS441, and PHE330 residues come after ARG445. In the case of Ligand4, there is a different order such that residue PHE330 is the most present followed by LYS331 and LYS441.

The H-bond formation shows that, during the production, GTP made nine H-bonds while Ligand4 made only four, as shown in Figure 9d,e.

The GTP molecule forms twice as many H-bonds compared to the Ligand4, this is due to the number of electronegative atoms available to form hydrogen bonds. GTP contains (Figure 4) phosphate, amine, and hydroxyl groups which provide more availability to form this kind of interaction against the hydroxyl and carbonyl groups present in the Ligand4.

#### 2.2.5. Molecular Mechanics Poisson–Boltzmann Surface Area (MM/PBSA)

**Figure 10:**
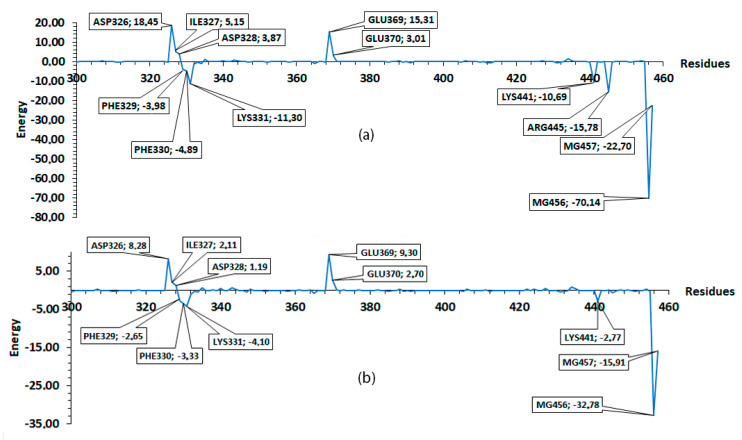
MM/PBSA average ligand binding free energies for 110 ns of simulation. Residues 300 to 457 were considered. (**a**) GTP and (**b**) Ligand4.

The MM/PBSA values presented are averages of the windows establish in this step. The values (with their standard deviation) include: −169.93 ± 4.03 for PleD–GTP; and −98.79 ± 0.43 for PleD–Ligand4. Figure 10 shows the average values of residues from different windows (20, 30, 40, 50, and 60) compared to previous results (Figure 5) for GTP. There exist a similarity, which indicates a tendency of residues like ASP326, ASP328, and GLU369, to have non-favorable interaction, while ARG445, LYS441, LYS331, PHE330, and PHE329 to have the major favorable contribution to the interaction with GTP. Thus, suggesting the importance of these residues in the binding pocket. In the case of Ligand4, the residues showed similar tendency as in the simulation of 10 ns, ASP326, ILE327, ASP328, GLU369, and GLU370 residues showed non-favorable interaction; whereas residues PHE329, LYS331, and LYS441 showed favorable interaction. In both cases, the magnesium atoms play an important role to the binding free energy.

The low standard deviation of the reported result at different windows is interpreted as high stability of the complexes. When comparing the two MM/PBSA results (first and second analysis), some variations in energy presented and emergence or disappearance of new residues, such as GLU370 or ASP343 for GTP, and LYS332 for Ligand4, is as a result of better conformational sampling obtained with more simulation time (110 ns). The result of MM/PBSA with short dynamic gives an idea of the overall interaction including the favorable or non-favorable interactions.

For the GTP results, where the major contribution for MM/PBSA binding free energy is the H-bond formation from ARG445, this residue is suggested as a key residue of ligand–protein interaction. The absence of this interaction with the rest of the ligands indicate a lower binding free energy in comparison with GTP. Both MM/PBSA results, as well as the one obtained by 10 ns and the average from the five windows, showed that the Ligand4 has good stability; thus, suggesting this molecule as a possible template for the design of new ligands that can surpass the GTP binding free energy and effectively inhibit the PleD protein.

Although the biosynthesis of *trans*-aconitic acid has been determined principally in soybean, maize, and wheat plants and some bacteria such as *Bacillus subtillis*, *Bacillus thuringiensis*, the enzymatic aconitate isomerase (AI; EC 5.3.3.7) activity was reported until now in the soil bacteria *Pseudomonas* sp. It has been associated, as it assimilates via the *trans*-aconitic acid into the bacterial tricarboxylic acid cycle, as a carbon source [46]. However, our goal in this research was to identify natural products as a potential starting point for hit-to-lead methodologies to obtain new inhibitors of the DGCs using as model PleD protein. On the other hand, it has been not reported that other bacteria possess the aconitate isomerase, and they could be used TAA as a carbon source too.

In this research, the binding free energy was calculated by MM/PBSA method and used to define a possible compound capable of inhibiting the PleD protein and, hence, blocking the formation of biofilms. TAA performed best among the 224,205 ligands studied. This compound is found in plants like *Zea mays*, *Triticum aestivum*, *Avena sativa*, *Brachiaria plantaginea*, and *Saccharum officinarum* [47]. Methods of extraction are described by Schnitzler et al. [48] and Kanitkar et al. [49]. In our results TAA does not surpass GTP binding free energy; however, it does not imply that it cannot be used. Our results suggest a good interaction with the PleD protein. Further optimization can be made to improve the results, using strategies like Fragment-Based Drug Discovery (FBDD) [50]. In addition, Fragment Molecular Orbital (FMO) [51,52] can be used to optimize the hit to make it more effective.

## 3. Materials and Methods

### 3.1. Data Collection

To search for antimicrobial agents from natural compounds, ZINC15 [53] database was chosen. ZINC15 is a database with public access established to allow ready access to compounds for VS. Molecules were downloaded in sdf format. ZINC15 has a natural product-related compounds database of about 224205. All records from naturals products were download and stored in a single file.

### 3.2. Virtual Screening

Structure-based VS with application of docking simulations was performed using ICM software package [34,54,55,56,57,58,59,60,61]. Virtual Ligand Screening in ICM was performed using the docking database downloaded from ZINC15 against PleD protein structure obtained from protein data bank PDB (PDB: ID 1W25) followed by an evaluation of the docked conformation with a binding-score function. The search space encompassed the catalytic domain, DGC (GGDEF) domain, which consists of a five-stranded central β-sheet surrounded by α-helices (Figure 2). Water molecules and ligands had been removed from the original crystal structure. ASP52 was phosphorylated by phosphate group, which was parameterized using procedures mentioned elsewhere [32]. A grid for space search was set up as reference using the GTPαS and Mg${}^{2+}$ from crystal structure, with the following dimensions in Å: center (x, y, z) = (52.1, 4.6, 76.2), dimensions (x, y, z) = (21.2, 17.9, 17.9). The docking simulation was then run at exhaustiveness of 5 and set to only output of the lowest energy pose.

### 3.3. Ligand Binding Free Energy Calculations

For the ligands, no geometry optimization was applied because the orientation obtained from the docking results were kept. Charge for ligands was calculated with ab initio methods using B3LYP and 6-31G as basis in GAUSSIAN09 software [62]. With the output file from gaussian09, charges and topology file (prepi and frcmod AMBER files, respectively) were prepared using antechamber and parmchk2 from Ambertools18 [63]. The charge file generated by antechamber, uses RESP charges; to improve results, these charges were replaced by the ones obtained from QM (B3LYB/6-31g) calculation.

### 3.4. System Preparation

The system consists of the protein–ligand complex embedded in a rectangular box with waters and ions. LEaP module of Amber was used for the preparation of all systems [63]. Initially, the mbondi2 parameter for PBRadii were set [64]. The ff14SB force field, which is an improvement of ff99SB, was used for the protein [65]. A total of 35 ligands with the best ICM energy score were chosen. The GAFF was used for the ligands [66] and all the systems were embedded in a box of size 100, 79, and 109 Å (approximately) for X, Y, and Z, respectively. TIP3P water type molecules [67,68] and Na${}^{+}$ ions were add to neutralize the charge.

### 3.5. Molecular Dynamics

For all systems, first, a minimization of only waters molecules was done. For this, the solute (protein and ligand) were restrained with a force of 100 kcal/mol-Å${}^{2}$. In this minimization, the maxcyc and ncyc as 50,000 and 1500 were established, respectively; and ntc was 1 (the shake was off). This was recommended during the minimization [63], so that a constant volume was established.

Afterward, a minimization of the entire system using maxcyc = 100,000 and ncyc = 1000 was done at constant volume. After energy minimization, the system was gradually heated until 300 K for 50 ps. In this part, the solute was restrained with a force of 2.0 kcal/mol-Å${}^{2}$ and, for the last step, the system was equilibrated for 5 nanoseconds (ns) and ntt = 3, ntp = 1, ntc = 2, and ntf = 2 were established with constant pressure. Finally, for the first analysis, we performed the production for 10 ns in NPT ensemble. For the second analysis of PleD–protein, PleD–GTP, and PleD–Ligand4 complexes, 100 ns was simulated. For PleD–GTP and PleD–Ligand4, the 100 ns production starts at end of the previous 10 ns, using the same conditions as the 10 ns production.

### 3.6. Molecular Mechanics Poisson–Boltzmann Surface Area (MM/PBSA) Calculation

The binding free energy was calculated using MM/PBSA script from AMBER package [69]. In the first analysis, a total of 200 snapshots were taken for MM/PBSA analysis. These snapshots are equal to the last 2 ns of simulation. Since Su et al. suggested that working with data of MD trajectories longer than 2 ns decreases the accuracy of the prediction [64]. The parameters igb = 2, inp = 1, and radiopt = 0 were used. The igb parameter is chosen depending on the mbondi parameters following the amber manual indications. The inp uses the solvent-accessible-surface area to correlate total non-polar solvation free energy and radiopt, thus indicating the default atomic radius of the input file [63]. In the calculation of MM/PBSA binding free energy, entropy was not considerate and this is due to the high computational cost of it. This contribution improves the result slightly or can decrease the effectiveness of the MM/PBSA method [70,71,72]. For the decomposition per residue of MM/PBSA result, the decomp general option was established with idecomp = 1 in the same input file. In the second analysis, we take the last 20, 30, 40, 50, and 60 ns of production and 200 snapshots, with the same condition as the first MM/PBSA calculation.

### 3.7. RMSD, RMSF, Radius of Gyration, and H-Bond

The RMSD, RMSF, Rg, and H-bond were calculated with cpptraj tool [43]. We used the entire production to carry out this analysis and the first frame was taken as reference for alignment in the RMSD and RMSF analysis.

## 4. Conclusions

Computational methods, using VS, play a significant role in drug discovery. The identification of drug interaction with target macromolecule can help to clarify the potential molecule’s mechanism of action. In our computational study, the binding free energy between PleD protein GTP binding site and ligands, was calculated through MM/PBSA to rank the best molecules obtained from VS of naturals products from ZINC15 database. We identified ARG445 as a key residue in the binding pocket. Residues LYS441, LYS331, PHE 330, and PHE329 present favorable interactions and GLU369 presents a common non-favorable interaction among the ligands studied. Our results suggest TAA as a potential starting point for hit-to-lead methodologies. The identified compound represents the most potential hit in the natural products library, which can be tested in vitro in order to optimize it to develop inhibitors of PleD.

## Figures and Tables

**Table 1:** ZINC15 ID and common name for best six MM/PBSA binding free energy score compounds.

Zinc15 ID	Name in this Work	Common Name
ZINC00895081	Ligand1	Citrate
ZINC03870145	Ligand2	Phosphoenolpyruvic acid
ZINC04028701	Ligand3	3-carboxy-2-(carboxymethyl)oxirane-2-carboxylate
ZINC04501392	Ligand4	*trans*-Aconitic acid
ZINC19336068	Ligand5	bis(4-hydroxy-2-oxo-2H-chromen-3-yl)acetic acid
ZINC27558828	Ligand6	2,3-Bis[(2E)-3-(4-hydroxyphenyl)-2-propenoyl]oxysuccinic acid

**Table 2:** Contribution of energies (kcal/mol) in MM/PBSA calculations.

Ligand	VDWAALS	EEL	EPB	ENPOLAR	Δ*G*
Ligand1	2.92 ± 4.30	−867.68 ± 21.67	766.94 ± 18.20	−1.64 ± 0.06	−99.46 ± 5.67
Ligand2	19.03 ± 4.87	−736.68 ± 24.60	615.55 ± 21.83	−1.22 ± 0.06	−103.32 ± 4.81
Ligand3	8.72 ± 5.14	−776.77 ± 38.28	650.15 ± 35.30	−1.67 ± 0.06	−119.58 ± 7.11
Ligand4	7.86 ± 4.83	−807.77 ± 33.78	680.01 ± 29.12	−1.43 ± 0.09	−121.33 ± 9.13
Ligand5	−2.20 ± 5.73	−686.56 ± 28.76	583.50 ± 27.10	−2.67 ± 0.13	−107.93 ± 8.92
Ligand6	−20.27 ± 4.39	−533.33 ± 19.94	456.46 ± 16.33	−3.84 ± 0.11	−100.98 ± 7.20
GTP	−10.86 ± 6.45	−1287.03 ± 35.53	1123.46 ± 29.55	3.49 ± 0.17	−178.09 ± 10.82

**Table 3:** The average ± standard deviation of RMSD for each PleD–ligand and PleD–GTP complexes in angstroms.

Ligand	RMSD	Difference between GTP and Ligand
Ligand1	2.261 ± 0.365	0.284
Ligand2	2.189 ± 0.336	0.212
Ligand3	2.165 ± 0.411	0.188
Ligand4	2.480 ± 0.255	0.503
Ligand5	2.506 ± 0.350	0.529
Ligand6	2.305 ± 0.389	0.328
GTP	1.977 ± 0.289	0

**Table 4:** Residues involved in the H-bond formation with percentage of how long this bond was present during the entire 10 ns of simulation.

Ligand	Residues Involve in the H-Bond Formation with the Major Contribution
Ligand1	LYS441 (54.20%), LYS331 (35.88%), PHE330 (7.11%), PHE329 (2.80%)
Ligand2	LYS441 (85.28%), PHE329 (9.37%), PHE330 (4.97%)
Ligand3	LYS441 (38.70%), PHE329 (24.17%), PHE330 (19.74%), LYS331 (17.39%)
Ligand4	LYS441 (52.68%), LYS331 (24.05%), PHE330 (19.28%), PHE329 (3.99%)
Ligand5	LYS441 (54.31%), PHE330 (35.45%), LYS331(9.31%)
Ligand6	LYS441 (36.13%), LYS331 (23.48%), PHE330 (17.81%), ASN334 (17.03%), LYS332 (4.77%)
GTP	ARG445 (39.05%), LYS331 (34.63%), LYS441 (17.35%), PHE330 (8.12%)

**Table 5:** Residues involved in H-bond formation with percentage of how long this bond was presented during 110 ns of simulation.

Ligand	Residues Involved in the H-Bond Formation with the Major Contribution
PleD–Ligand4	PHE330 (9.7%), LYS331 (6.01%), LYS441 (5.69%)
PleD–GTP	ARG445 (41.44%), LYS331 (15.14%), LYS441 (12.07%), PHE330 (11.95%)

## Supplementary Materials

The following are available online.

## Abbreviations

The following abbreviations are used in this manuscript:

EPS	Extracellular polymeric substances
c-di-GMP	Cyclic diguanylate
DGC	Diguanylate cyclases
PDEs	Phosphodiesterases
GTP	Guanosine-5’-triphosphate
TTA	*trans*-aconitic acid
VS	virtual screening
MD	molecular dynamics
MM/PBSA	Molecular Mechanics Poisson-Boltzmann Surface Area
MMFF	Merck Molecular Force Field
RMSD	Root Mean Square Deviation
RMSF	Root Mean Square Fluctuation
Rg	Radius of gyration
H-bond	Hydrogens Bond
