# Supplementary material for: Searching Hit Potential Antimicrobials in Natural Compounds Space against Biofilm Formation
Source: Molecules. 2020 Nov 16;25(22):5334. doi: 10.3390/molecules25225334 (PMC7696173; doi:10.3390/molecules25225334)
Supplement: Supplementary file 1 [file molecules-25-05334-s001.pdf]

# Supplementary Materials: Searching Hit Potential Antimicrobials in Natural Compounds Space Against Biofilm Formation

**Roberto Pestana-Nobles [1,‡], Jorge A. Leyva-Rojas [1,‡] and Juvenal Yosa [1,‡]***

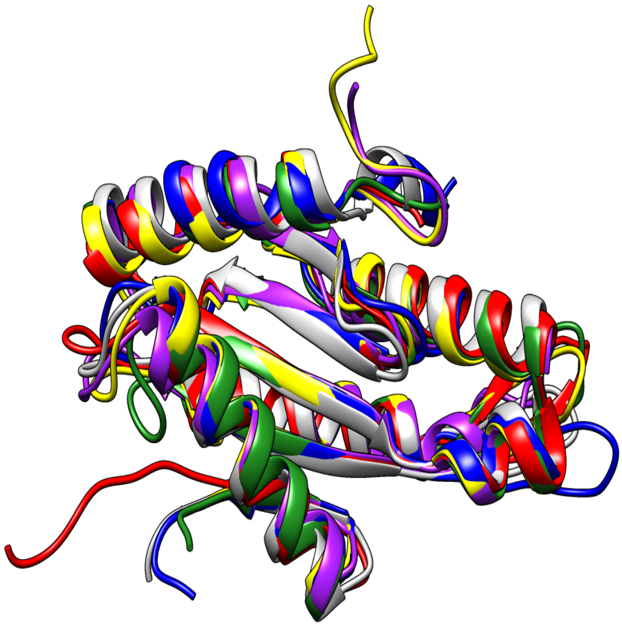

**Figure S1.** Alignment of five crystal structure of GGDEF domain compared with PleD for *Vibrio cholerae* (gray), E.coli DosC (blue), *Marinobacter aquaeolei* (yellow), *Pseudomonas aeruginosa* (green), and *P. fluorescens* (purple). All of them form a biofilm. As is shown the structural domain is well conserved. GTP has a high affinity for the active site of all GGDEF domain. Here, we assume that the active site for all the GGDEF domains in bacteria, binds GTP molecule similarly. In such a case, the hit that can be found in the natural products library the natural products library will be bind also similar to all GGDEF domains in bacteria. The RMSD between them is 0.915 Å, this structure was obtained using the needleman-Wunsch matrix BLOSUM62 algorithm.

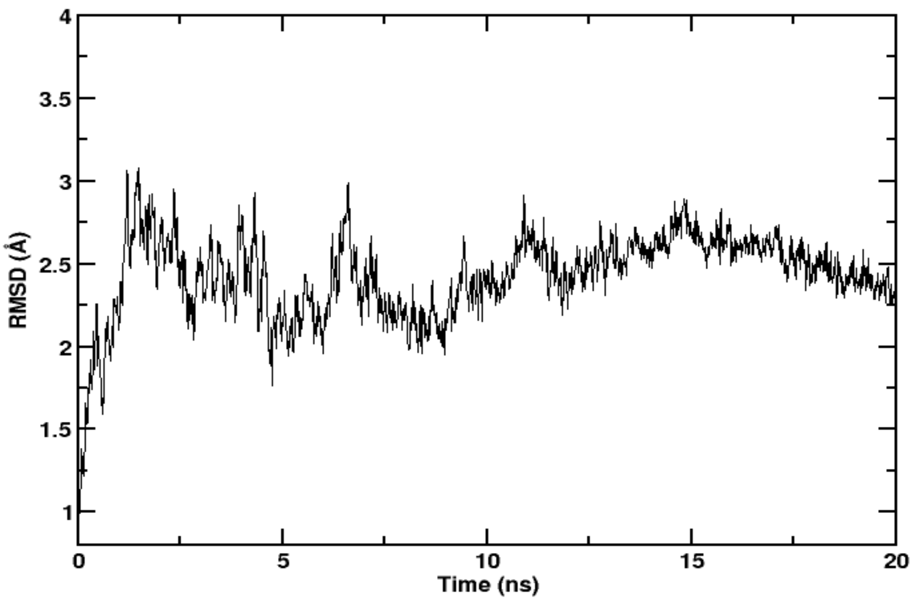


**Figure S2.** Root mean square deviation RMSD for the first 20 ns of MD simulation for average structure calculation.

**Table S1.** ICM energy score for the best 100 hit from virtual screening.

**Begin of Table**

| ZINC15 id | Score (kcal/mol) |
|---|---|
| GTP | -27.64327 |
| ZINC04028703 | -47.139771 |
| ZINC38145808 | -45.671547 |
| ZINC19336068 | -44.979202 |
| ZINC19336068 | -44.134228 |
| ZINC14824069 | -42.93782 |
| ZINC72131066 | -42.670437 |
| ZINC19336068 | -41.267788 |
| ZINC01531039 | -41.213696 |
| ZINC61389537 | -40.760124 |
| ZINC13385611 | -40.739395 |
| ZINC08764797 | -40.055969 |
| ZINC85994780 | -39.867146 |
| ZINC15956889 | -39.779278 |
| ZINC20112504 | -39.353798 |
| ZINC20112501 | -38.948174 |
| ZINC70706013 | -38.285667 |
| ZINC19336068 | -38.136402 |
| ZINC08765165 | -38.005142 |
| ZINC70665805 | -37.436745 |
| ZINC04061910 | -37.341373 |
| ZINC04028701 | -37.196304 |

**Continuation of Table S1**

| ZINC15 id | Score |
|---|---|
| ZINC03870145 | -37.095665 |
| ZINC20112413 | -36.982067 |
| ZINC85489989 | -36.273315 |
| ZINC35365918 | -36.247917 |
| ZINC85509549 | -35.961651 |
| ZINC27558828 | -35.729488 |
| ZINC35363853 | -35.638927 |
| ZINC13783218 | -35.60701 |
| ZINC02154275 | -35.538185 |
| ZINC12890057 | -35.364208 |
| ZINC03984661 | -35.360455 |
| ZINC85568960 | -35.339859 |
| ZINC95099067 | -35.153324 |
| ZINC31156942 | -34.981937 |
| ZINC20112388 | -34.671345 |
| ZINC08214655 | -34.58791 |
| ZINC15957926 | -34.518906 |
| ZINC04501392 | -34.423988 |
| ZINC04501392 | -34.37841 |
| ZINC49543170 | -34.348434 |
| ZINC00895180 | -34.218311 |
| ZINC40312960 | -34.154789 |
| ZINC06624586 | -34.142925 |
| ZINC49543372 | -34.080452 |
| ZINC40312777 | -33.969444 |
| ZINC40312780 | -33.945049 |
| ZINC40312441 | -33.902905 |
| ZINC00895081 | -33.780499 |
| ZINC13460583 | -33.716133 |
| ZINC20611409 | -33.608517 |
| ZINC11867541 | -33.59919 |
| ZINC85507641 | -33.556717 |
| ZINC03870145 | -33.400269 |
| ZINC01628282 | -33.386013 |
| ZINC04029191 | -33.353973 |
| ZINC95913918 | -33.329704 |
| ZINC85506516 | -33.233322 |
| ZINC00895212 | -33.085876 |
| ZINC13548192 | -33.034981 |
| ZINC20112845 | -33.005642 |
| ZINC96222540 | -32.85162 |
| ZINC20112987 | -32.810581 |
| ZINC00895081 | -32.805515 |
| ZINC59777472 | -32.789963 |
| ZINC30726567 | -32.767242 |
| ZINC85541484 | -32.749729 |
| ZINC00057646 | -32.733398 |
| ZINC40312957 | -32.714645 |

**Continuation of Table S1**

| ZINC15 id | Score |
|---|---|
| ZINC98368979 | -32.698872 |
| ZINC00895081 | -32.678986 |
| ZINC04164596 | -32.590797 |
| ZINC38145807 | -32.561115 |
| ZINC00895081 | -32.549793 |
| ZINC36368084 | -32.516823 |
| ZINC12890057 | -32.491215 |
| ZINC85507538 | -32.419991 |
| ZINC49543329 | -32.386856 |
| ZINC40313216 | -32.314423 |
| ZINC20112385 | -32.311005 |
| ZINC09033627 | -32.244923 |
| ZINC06112681 | -32.221901 |
| ZINC59587150 | -32.219753 |
| ZINC20611262 | -32.195625 |
| ZINC40313128 | -32.163731 |
| ZINC95098987 | -32.112232 |
| ZINC38545590 | -32.107368 |
| ZINC12296571 | -32.065216 |
| ZINC00895081 | -32.045967 |
| ZINC85548977 | -31.981853 |
| ZINC95912584 | -31.889652 |
| ZINC85568565 | -31.822807 |
| ZINC08790482 | -31.818468 |
| ZINC70706327 | -31.789642 |
| ZINC96222502 | -31.707294 |
| ZINC95099065 | -31.684559 |
| ZINC20113196 | -31.678776 |
| ZINC20611063 | -31.675303 |
| ZINC96116292 | -31.64535 |
| ZINC12297114 | -31.619473 |

End of Table

**Table S2.** Result of MM/PBSA calculation from the best 35 ligands from virtual screening from the 10 ns simulation.

| Zinc15 id | $\Delta G$ |
|---|---|
| ZINC00895081 | -99.4252 |
| ZINC02154275 | -26.0056 |
| ZINC03870145 | -103.3190 |
| ZINC03984661 | -19.7298 |
| ZINC04028701 | -119.6044 |
| ZINC04028703 | -86.3944 |
| ZINC04061910 | -65.4872 |
| ZINC04501392 | -121.3924 |
| ZINC06624586 | -22.4335 |
| ZINC08764797 | -19.9280 |
| ZINC08765165 | -37.7270 |
| ZINC13460583 | -30.0101 |
| ZINC13783218 | -53.0186 |
| ZINC15956889 | -12.8434 |
| ZINC15957926 | -15.4614 |
| ZINC19336068 | -107.8820 |
| ZINC20112413 | -86.6941 |
| ZINC20112501 | -78.3754 |
| ZINC20112504 | -76.4244 |
| ZINC27558828 | -101.0158 |
| ZINC35363853 | -13.7876 |
| ZINC35365918 | -17.0339 |
| ZINC38145808 | -42.6426 |
| ZINC40312441 | -37.3111 |
| ZINC40312777 | -49.1184 |
| ZINC40312780 | -34.0688 |
| ZINC40312960 | -46.9143 |
| ZINC49543170 | -42.5067 |
| ZINC49543372 | -32.4974 |
| ZINC61389537 | -31.3838 |
| ZINC70665805 | -16.5726 |
| ZINC70706013 | -48.3709 |
| ZINC72131066 | -19.3442 |
| ZINC85509549 | -32.3730 |
| ZINC85568960 | -55.4133 |
| ZINC95099067 | -33.2993 |
